# Supplementary material for: Chromothripsis during telomere crisis is independent of NHEJ, and consistent with a replicative origin
Source: Genome Res. 2019 May;29(5):737–49. doi: 10.1101/gr.240705.118 (PMC6499312; doi:10.1101/gr.240705.118)
Supplement: Supplemental Material [file supp_gr.240705.118_Supplemental_file_1.zip › contigs/annotated_contigs/DB51/contig.2.DB51_length_722_mean_cov_5.53462603878.docx]

**DB51_length_722_mean_cov_5.53462603878**

```
GAGAACTCTAGGTGGAATGTGGTAACTTTTGCCAGGGCAGAGGGAACACCTACTAATAGGTACTTCATTTGCACCACCAGAGATTGGCA
 >chr6:12299105-12299468 + E=1e-206
TCTTTTTTGATGGATCCACTGGCTTTGATACTGCCTGTACTCCCCCAAAACACAGCTTGGGTATTGGACTAATCTAGAGCTCCCTCAGG

AGAACTCTTGCTGACATTAAGAAAGAGCAACATTTTGTCTTTCCAGGTGAAAATCCAAGGCCCAAAAAGGGAGGTGACTCACCTAAGAT

CACAGAAGGAGCTGTAGCATCTCTGGAGCCTGAACACTTAAGTTAAGCACGACTATTTCAGCCAGAGGGCCCCTGGAGGTCTGCAGAAA

CAGATGC|TGTTTCT|GTTTGGGGTGAGCCTTTCTTAGGAGCTCCATTAGCCAACAGCCAAGATACCTCATTTTGCACTCACTTCACTT
               >chr6:158472555-158472837 + E=7e-158
GTGACCAAAGTGTCTTAGGACCAAAAGGGATTTGGGACTTTGTTGAACTGTTTATATAAAAAAAACCTGGCTGGGCACAATGGCTCAAG

CCTGTAATCTCAGTACTTTGAGAGGCCGAGGTGGGTGGATCACTTGAGCCCGGGAGTTCAAGACCAACGTGGGCAGCATGGCGAAACCC

CATCTCTACAAAAAATACAAAAAAAAAAAAA|TGGCGAGGGGTGGGGGGGTACCGTTGTGGGCCGGCCACCTCGGGGGGCGGGGGGGGG

GGGGGGATTGAAA
```
